# Dataset of human-single neuron activity during a Sternberg working memory task

**DOI:** 10.1038/s41597-024-02943-8

**Published:** 2024-01-18

**Authors:** Michael Kyzar, Jan Kamiński, Aneta Brzezicka, Chrystal M. Reed, Jeffrey M. Chung, Adam N. Mamelak, Ueli Rutishauser

**Affiliations:** 1 Department of Neurosurgery, Cedars-Sinai Medical Center, Los Angeles, CA USA; 2 Center of Excellence for Neural Plasticity and Brain Disorders: BRAINCITY, Nencki Institute of Experimental Biology, Polish Academy of Sciences, Warsaw, Poland; 3 Institute of Psychology, SWPS University of Social Sciences and Humanities, Warsaw, Poland; 4 Department of Neurology, Cedars-Sinai Medical Center, Los Angeles, CA USA; 5 Center for Neural Science and Medicine, Department of Biomedical Sciences, Cedars-Sinai Medical Center, Los Angeles, CA USA; 6 Division of Biology and Biological Engineering, California Institute of Technology, Pasadena, CA USA

**Keywords:** Cognitive neuroscience, Working memory

## Abstract

We present a dataset of 1809 single neurons recorded from the human medial temporal lobe (amygdala and hippocampus) and medial frontal lobe (anterior cingulate cortex, pre-supplementary motor area, ventral medial prefrontal cortex) across 41 sessions from 21 patients that underwent seizure monitoring with depth electrodes. Subjects performed a screening task (907 neurons) to identify images for which highly selective cells were present. Subjects then performed a working memory task (902 neurons), in which they were sequentially presented with 1–3 images for which highly selective cells were present and, following a maintenance period, were asked if the probe was identical to one of the maintained images. This Neurodata Without Borders formatted dataset includes spike times, extracellular spike waveforms, stimuli presented, behavior, electrode locations, and subject demographics. As validation, we replicate previous findings on the selectivity of concept cells and their persistent activity during working memory maintenance. This large dataset of rare human single-neuron recordings and behavior enables the investigation of the neural mechanisms of working memory in humans.

## Background & Summary

Working memory (WM) plays a crucial role in various cognitive functions, including decision-making, attention, and problem-solving^1^. Current models of WM suggest that one mechanism by which memoranda can be maintained is through persistent activity^2–4^. It is thought that a distributed network of brain areas supports WM maintenance, as indicated in prior studies in nonhuman primates^5–12^. However, direct translation of findings from animals to humans is challenging due to the inherent differences^13,14^ in brain organization and cognitive abilities. In rare clinical circumstances, it is possible to invasively record electrophysiological signals from humans at the single-neuron resolution level using depth electrodes while subjects are performing cognitive tasks. These opportunities have provided invaluable insights into the mechanisms of human working memory^15–19^. This work has revealed that within the human medial temporal lobe (MTL), a subset of highly selective concept cells can remain persistently active for several seconds during working memory maintenance^15^. We have further shown that the activity of these persistently active cells allows decoding of working memory content and predicts working memory quality and accuracy, thereby indicating that these cells are part of the neuronal substrate of WM maintenance^15,20,21^. Remarkably, in contrast to the MTL, there were few selective concept cells in the medial frontal cortex (MFC) areas we recorded from. Instead, we have characterized maintenance and probe cells in the MFC, whose activity are not WM-content selective but instead is predictive of working memory quality alone. These findings indicate that the role of the MFC in WM maintenance is control and monitoring rather than maintaining memory content^22,23^. This data descriptor accompanies a public release of this cumulative dataset in the Neural Data without Borders (NWB) format^24^ hosted on the DANDI data archive. We chose NWB as the data format for this release because it is a standardized format well suited for cellular-level data around which a mature ecosystem of APIs, data archives, analysis software, policies, and cloud-based compute platforms has recently emerged. While significant work was required to convert the data into this relatively complex format (containing all needed data in a single file), we chose to pursue this route given these significant benefits. We note that in contrast, iEEG-BIDS^25^ specifies a directory structure rather than a data format. We chose DANDI as our data archive due to its close integration with NWB, the automatic and extensive validation process that is enforced before upload, and the suitability of enabling large scale meta-analyses that DANDI enables^25^.

**Fig. 1:**
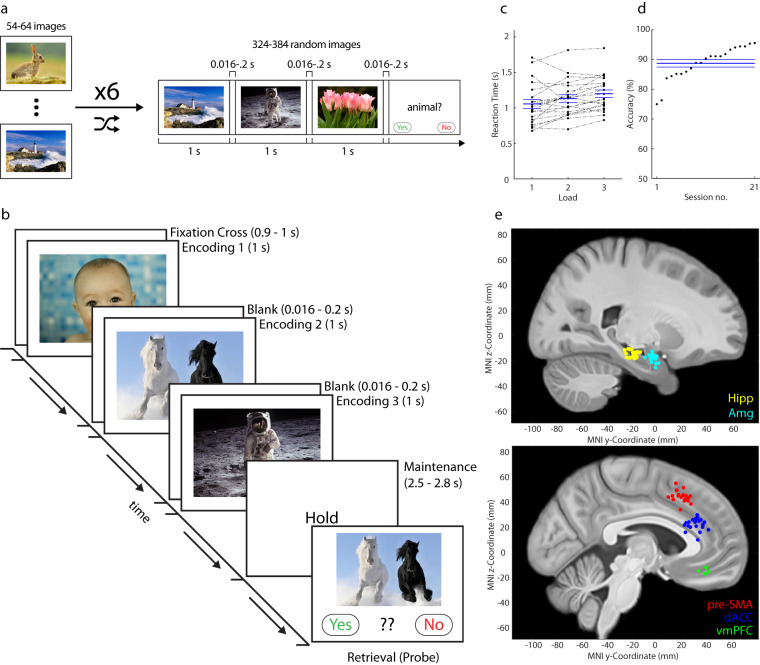
Task, behavior, and electrode localization. (**a**) Screening task. 54–64 images were shown six times each in random order. The subject was asked if the preceding picture was a person/landscape/animal every few images (randomized). (**b**) Sternberg task. After the presentation of a fixation cross (baseline), the subject was sequentially shown 1–3 images (encoding), followed by a variable delay during which the screen was blank (maintenance). After this, a probe image was shown, and subjects indicated if the probe image belonged to the set of images presented in the encoding period. (**c,d**) Behavior. (**c**) Median reaction time across loads (relative to probe stimulus onset). A dashed line links performance for each load in a session. (**d**) Accuracy, rank ordered. In **c**,**d**, the mean and s.e.m of each group are denoted by a thick blue line and lighter blue lines, respectively. (**e**) Locations of recording sites in Montreal Neurological Institute’s MNI152 coordinates superimposed onto the California Institute of Technology’s CIT168 T1w brain atlas (see MRI Processing and Electrode Localization). Locations are color-coded by location site (yellow, Hippocampus (Hipp); cyan, Amygdala (Amg); red, pre-SMA; blue, dACC; green, vmPFC). Each dot is a recording bundle, projected onto a single hemisphere for illustration purposes.

To investigate the mechanisms underlying WM, we employed a modified version of the well-established Sternberg^26,27^ task that utilizes images as stimuli (Fig. 1b). We have used this identical task across multiple studies^15,20,28^, the data for which are all included in the present dataset. Our dataset includes recordings obtained during the Sternberg task and a preceding screening task (Fig 1a) in both the medial temporal lobe (MTL) and the medial frontal lobe (MFL) regions. In total, the dataset comprises electrophysiology recordings of 902 neurons from Sternberg sessions and 907 neurons from screening sessions and encompasses data from 21 patients. During the Sternberg task, participants were presented with a set of images for memorization. Each trial consisted of 1–3 images, followed by a maintenance period and a probe image. Participants then indicated whether the probe image belonged to the initial set of images. The 1–3 images were pseudo-randomly selected from the 5 images that elicited the highest selective response in the preceding screening task (Fig. 1a). The stimuli used in the task encompassed a diverse range of content, including images of animals, objects, people, and other complex natural scenes. This allowed for the investigation of neural responses to different types of visual stimuli. In addition to single-neuron recordings, the dataset includes behavioral data, providing a comprehensive view of participants’ performance during the task. For technical validation, we include spike sorting quality metrics as well as replication of prior results regarding the activity of concept cells during WM maintenance. Potential applications for the use of this dataset include the testing of predictions made by working memory models^1,29^, and analysis of population-level neuronal dynamics^30–32^ that necessitates hundreds of neurons to be implemented.

## Methods

The methods used to acquire and process this dataset have been previously published^15,20^ but are abbreviated here for convenience.

### Subjects

In total, 41 recording sessions were completed across 21 patients with intractable localization-related epilepsy (Table 1). Patients underwent intracranial monitoring using depth electrodes for seizure localization. Electrode locations were solely based on clinical criteria. Voluntary participation in this study was offered to all patients that underwent monitoring for seizures with depth electrodes at participating study sites. Patient data has been de-identified, with each patient identified only by a unique patient identifier string. The identifier is comprised of a numerical patient ID, followed by an abbreviation of the recording institution (CS: Cedars-Sinai Medical Center, HMH: Huntington Memorial Hospital) (e.g., P42CS). All patients volunteered to participate in this study and provided written informed consent. Participants’ age ranged from 17 to 71 and in cases where subjects were under 18 years of age, informed consent was also obtained from the participant’s legal guardian. All protocols were approved by the Institutional Review Boards of Cedars-Sinai Medical Center (IRB: 13369), Huntington Memorial Hospital (IRB: 27132), and the California Institute of Technology (IRB: 16-0692 F).

**Table 1:** Patients.

Subject ID	Native Subject ID	Age	Sex	Epilepsy Diagnosis	Screening	Sternberg
1	P42CS	25	F	Not Localized	Yes	Yes
2	P43CS	42	F	Left Hippocampus	Yes	Yes
3	P44CS	53	F	Right Medial Temporal (Hippocampal)	Yes	Yes
4	P47CS	32	M	Right Mesial Temporal	Yes	Yes
5	P48CS	32	F	left medial temporal (Amyg/Hipp)	Yes	Yes
6	P31CS	32	M	Left Temporal Neocortical	Yes	Yes
7	P32CS	19	M	Not Localized (Generalized)	Yes	Yes
8	P33CS	44	F	Right Mesial Temporal	Yes	Yes
9	P34CS	70	M	Bilateral Mesial Temporal	Yes	Yes
10	P35CS	63	M	Left Temporal Neocortical	Yes	Yes
11	P36CS	45	M	Right Hippocampus	Yes	Yes
12	P37CS	33	F	Right Hippocampus	Yes	Yes
13	P39CS	26	M	Right insula, No Temporal Involvement	Yes	Yes
14	P39CS_2	26	M	Right insula, No Temporal Involvement	Yes	Yes
15	P40CS	25	M	Right Motor Cortex	Yes	Yes
16	P48HMH	54	M	Left Temporal (Amygdala)	Yes	Yes
17	P49HMH	54	F	Right Amygdala and Hippocampus	Yes	Yes
18	P51HMH	24	M	Bilateral Frontal and Temporal	Yes	Yes
19	P47HMH	20	M	Right Amygdala	No	Yes
20	P49CS	24	F	Left Mesial Temporal (Amygdala)	Yes	Yes
21	P51CS	17	M	Not Localized (No Seizures)	Yes	Yes

A list of all subjects in the dataset. The age of the subject is at the time of recording. The diagnosis listed (focal epilepsy) was determined from depth electrode monitoring at the time of recording. All subjects have at least one recording session for the WM task. Screening sessions are available for all subjects except for ‘P47HMH’, for whom screening was not performed.

### Electrodes and data acquisition

Recordings were performed using commercially available FDA-approved hybrid macro-micro depth electrodes (Ad-Tech Medical). The hybrid macro electrode consists of 4–8 cylindrical platinum-iridium ECoG electrodes spaced at 5 mm intervals along a hollow polyurethane shaft. The microwires are a bundle of eight wires (40um diameter) threaded through the macroelectrode shaft^33,34^ to the target area. Each microwire was locally referenced to a common microwire in the same bundle, allowing for the recording of activity from seven microwires in each area. The continuously acquired raw signal was recorded broadband (0.1–9000 Hz filter) and sampled at 32 kHz using either a Neuralynx ATLAS or Neuralynx Cheetah System (Neuralynx Inc.). Channels exhibiting inter-ictal epileptic activity at the time of recording were excluded from analysis.

### Spike detection and sorting

Spike detection and sorting were performed with OSort, an open-source semiautomatic template-matching algorithm^35^. First, the signal from each channel (i.e., microelectrode) was filtered with a zero-phase lag filter in the 300–3000 Hz band. Spikes were detected using the energy power method, consisting of threshold crossings of the energy signal computed by convolving the filtered raw signal with a kernel of approximate width of an action potential^35,36^. To assess the quality of identified clusters, the following criteria were used: (*i*) firing rate stability, (*ii*) waveform amplitude stability, (*iii*) interspike interval (ISI) distribution, and (*iv*) violations of the refractory period. Similar-looking clusters were merged. All clusters that passed the given criteria were stored and are provided as a list of timestamps. Spike sorting was done independently for the two tasks (Screening, Sternberg).

### MRI Processing and electrode localization

Electrodes were localized based on pre-operative structural MRIs and post-operative MRIs and/or CTs. Brains were extracted from the pre and post-operative scans^37^, and the post-operative scan was aligned to the pre-operative scan using Freesurfer’s mri_robust_register^38^. If a post-operative MRI was unavailable, the post-operative CT scan was co-registered with a rigid (6 DOF) transform using the BRAINSFit^39^ program to the pre-operative MRI using 3DSlicer^40^. A forward mapping of the pre-operative scan to the CIT168 template brain^41^ was computed using a concatenation of an affine transformation and a symmetric image normalization (SyN) diffeomorphic transform from the ANTs suite of programs^42^. This transform was then applied to the post-operative scan. This resulted in a post-operative scan overlayed onto the MNI152-registered version of the CIT168 template brain^41^. Freesurfer’s Freeview program was then used to mark the electrodes as point sets to determine the locations of the microwire tips. The electrode positions are provided in 3D but are projected onto the 2D sagittal plane for visualization only (Fig. 1e).

### Psychophysics

The tasks have been previously described but are summarized here for convenience^15^.

Subjects performed two tasks: a screening task, followed by a working memory task (the latter is referred to as the “Sternberg task”).

Screening Task: Images to be shown were selected based on a participant’s interest, and 54–64 of these images were shown six times each in randomized order for 1 s each, with an interstimulus interval of 0.016–0.2 s (Fig. 1a). Images shown were approximately 9° × 9° in size on the screen. Every few trials (randomized), a control question was asked that was related to the previously displayed image (i.e., “Did the last image present a person/landscape/animal?”). After the screening task, the data was rapidly analyzed to determine the five images that elicited the best responses, using an F-statistic computed by a one-way 1xN ANOVA (n = number of images, i.e. 54–64) for the mean response in a 200–1000 ms window following stimulus onset, with the image as a factor (See Identification: Concept Cells). If less than five neurons showed significantly selective responses (p< 0.05), the remaining images were selected based on the strongest non-selective responses. The five chosen images were then used for the Sternberg task.

Working Memory Task: A modified version of the Sternberg^27^ task was used that employed images rather than the traditional digits as the memorization material (Fig. 1b). For each trial, a fixation cross was shown for 900–1000 ms, followed by the sequential presentation of the 1–3 images to be memorized (‘encoding’) in the given trial. Each image was shown for 1 s, followed by a blank screen for a randomized period of 1–200 ms. Images shown were approximately 9° × 9° in size. Subjects were instructed to memorize the 1–3 images in each trial. The terms’ encoding 1’, ‘encoding 2’, & ‘encoding 3’ refer to the 1–3 images presented in each trial. After the encoding stage, there was a maintenance (delay) period of 2.5–2.8 s, during which only the word “hold” was shown on the screen. At the end of the delay period, a probe stimulus was displayed, and subjects were asked to indicate whether the probe stimulus was one of the immediately preceding 1–3 images or not. Participants responded by pressing a green or red button on an external response box (RB-844, Cedrus Inc.), the color corresponding to ‘yes’ and ‘no’ being shown at the top of the screen for each probe trial. The locations of the ‘yes’ and ‘no’ buttons were switched halfway through the experiment. Subjects were asked to respond as quickly as possible. The probe stimulus was present until subjects pressed one of the two buttons. Each session consisted of 108 or 135 trials, depending on the task variant, and the images were displayed in pseudorandom order. The pictures shown to each participant were unique and were determined based on the results of a screening task that occurred 2–3 hours prior (as described above), except for one participant who did not undergo the screening procedure (see Table 1).

The screening and Sternberg task were run on a notebook computer, and both were implemented in MATLAB using the Psychophysics Toolbox^43^. Subject responses and stimulus onset/offset markers were sent to the acquisition system via TTLs sent over a parallel port (Tables 2–3**)**.

**Table 2:** Sternberg task event markers.

Event TTL ID	Description
61	Start of Experiment
11	Fixation Cross
1	Picture #1 Shown
2	Picture #2 Shown
3	Picture #3 Shown
5	Transition Between Picture Presentation
6	End of Encoding Sequence/Start of Maintenance Period
7	Probe Stimulus Onset
8	Subject Response
60	End of Experiment

Event markers (“TTLs”) used in the Sternberg task. For each trial, a fixation cross (11) was presented to the subject. After which, picture #1 (1) was shown. This was followed either by a transition (5) between images or an end (6) of the encoding sequence, depending on whether more images (2,3) were shown in the trial. After the maintenance period (6), a probe image (7) was shown. The subject then responded (8) by pressing a button on a response pad. The ‘description’ field in \acquisition\events of the provided NWB files also contains the information listed in this table.

**Table 3:** Screening task event markers.

Event TTL ID	Description
61	Start of Experiment
1	Start Picture Presentation
3	End Picture Presentation
4	Subject Response
60	End of Experiment

Event markers (“TTLs”) used in the screening task. Subjects were shown a set of images six times in a randomized order. Subjects were shown (1) each image for 1 s, at which point the presentation of that image ceased (3). As a control, after a randomized number of trials, subjects responded (4) to a question related to the preceding image (i.e., “Did the last image present a person/landscape/animal?”). The ‘description’ field in \acquisition\events of the provided NWB files also contains the information listed in this table.

### Cell selection: concept cells

Single units were considered visually selective concept cells^44^ if their firing rates significantly covaried as a function of picture identity in a 200–1000 ms window following stimulus onset. Concept cells were identified (P < 0.05) using a permuted one-way ANOVA with *x* groups, with *x* the number of unique images presented to the subject in a session. For the screening task, *x* varied between 54–64. For the Sternberg task, *x* was 5. If a cell passed the ANOVA test, we in addition tested whether the response to the image with the maximal response was significantly larger than that for all the other images (P < 0.05, permutation t-test). A cell that satisfied both criteria was considered a concept cell for the Sternberg task. We note that by ‘concept cell’ we refer to cells that are visually selective. We did not assess or require selectivity for the same concept in other modalities (text, auditory) as is sometimes required for a cell to qualify as a concept cell in some other studies^44,45^.

### Cell selection: maintenance cells

A single unit was considered as a maintenance cell if its response increased significantly in the maintenance period relative to the baseline period. This increase must be invariant to the identity of the stimulus that was held in memory. Maintenance cells were identified by comparing the mean firing rate during the maintenance period (0–2500 ms) to the firing rate during the presentation of the fixation cross at the start of a trial (500 ms) (P < 0.05, permutation test). If a concept cell also qualified to be a maintenance cell using this criterion, an additional test was run to assure that the maintenance activity for all non-preferred stimuli was significantly higher than during the baseline. This second requirement ensures that strong concept cells are not automatically also classified as maintenance cells.

### Cell selection: probe cells

A single unit was considered as a probe cell if its response was significantly higher during the probe period relative to both the encoding and maintenance periods. Probe cells were identified using two separate permutation t-tests (P < 0.05) between the firing rates following probe onset (200–1000 ms) and the firing rates in the encoding (200–1000 ms) and maintenance (0–2500 ms) periods.

## Data Records

All data^46^ associated with this release is available on the DANDI Archive (dandiset #469), a BRAIN Initiative-supported archive for publishing and sharing neurophysiology data. Two NWB files are provided for each subject, one for each task (Screening, Sternberg). The filenames are of the form ‘sub-[Subject ID]-ses-[Task ID].’ The task ID indicates the type of session performed, (1: Screening (SC), 2: Sternberg (SB)). Each file contains an ‘identifier’ field containing a string of the form ‘TaskID_subjectID_nativeSubjectID’. Task ID equates to SB (Sternberg) or SC (Screening), Subject ID is a unique index into the internal data structures (see Table 1), and Native Subject ID is the patient identified as described above.

### Structure of the NWB File

Each file contains all the data available for a single task for a given subject. The file format is the Neurodata Without Borders: Neurophysiology 2.0 (NWB:N) format. NWB:N^24,47^ is a standardized environment for disseminating neurophysiological data. NWB files can be read using standard APIs that are available for different operating systems and programming languages.

**Fig. 2:**
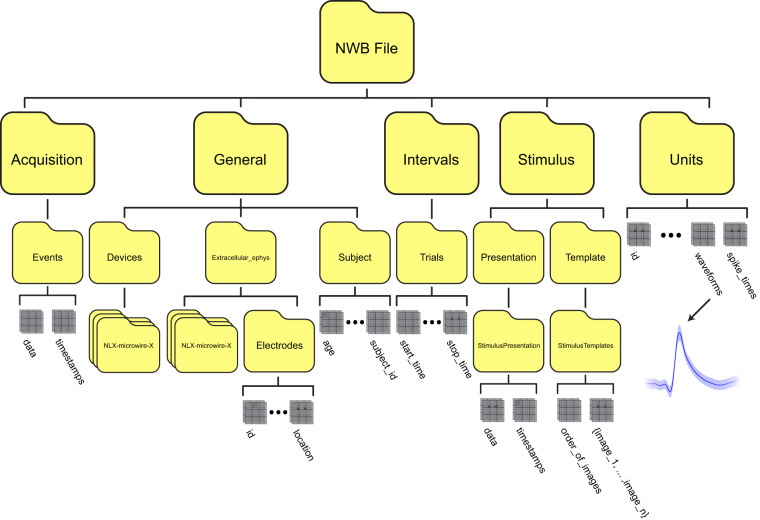
File structure. The organizational scheme for storing human single-neuron data in NWB format. The top-level groups of each file are Acquisition, General, Intervals, Stimulus, & Units. Each top-level group can be indexed into the subset of data desired for further analysis.

An NWB file comprises groups (like a directory) that store different subsets of data with pre-defined names and data types. For our dataset (Fig. 2), we used the containers ‘general’ (metadata for subjects & recording devices), ‘acquisition’ (raw data streams), ‘intervals’ (trial timestamps and trial-specific behavioral flags), ‘stimulus’ (images presented to the subject), and ‘units’ (spike times of putative single units). The ‘description’ field in each container was utilized to give further information on the structure and usage of the data. At the top level of each NWB file, additional meta data is stored in the fields ‘identifier,’ ‘file_create_date,’ ‘session_description,’ ‘session_start_time,’ & ‘timestamps_reference_time.’ All data is stored as prescribed by the NWB standard.

### NWB File content: general group

The \general group contains metadata. At the top level of \general, metadata about the experiment is stored in the fields ‘experiment_description,’ ‘experimenter,’ ‘institution,’ ‘keywords,’ ‘lab,’ ‘notes,’ ‘related_publications,’ ‘session_id,’ and ‘source_script.’ Electrode information is provided in general\extracellular_ephys\electrodes as a column-based table, with one row per electrode. The electrode ID, the filter applied, the group name, the brain area, and the x/y/z coordinates in MNI space are specified for each electrode. Each channel also contains a soft link to an ElectrodeGroup container in \general\extracellular_ephys. This subgroup then links to a Device subgroup in general\devices that indicates the device used for signal acquisition, which here is the Neuralynx Inc. amplifier (“Neuralynx-Atlas” or “Neuralynx-Cheetah”). Electrode IDs are referenced in other parts of the NWB file when necessary (see **NWB File Content: Units Group**). Additionally, the custom field ‘origChannel’ in the electrodes table denotes the channel number of the native recording system. Lastly, subject metadata is provided in the general\subject subgroup (subject’s age, sex, species, and subject ID).

### NWB File content: acquisition group

The \acquisition group contains the raw stream of TTLs to align the behavior with the neural data. The acquisition\events subgroup contains the stream of TTL event markers and the timestamps at which they occurred. The following TTL values were used for Sternberg sessions (Table 2): Start of Experiment (61), Fixation Cross Onset (11), Picture #1 Shown (1), Picture #2 Shown (2), Picture #3 Shown (3), Transition Between Picture Presentation (5), End of Encoding Sequence/Start of Maintenance Period (6), Probe Stimulus Onset (7), Subject Response (8), & End of Experiment (60). The following TTL values were used for screening sessions (Table 3): Start of Experiment (61), Picture Presentation (1), End Presentation (3), Subject Response (4), & End of Experiment (60).

### NWB File content: intervals group

The \intervals group specifies the trials in the session. Data is contained as a column-based table in the subgroup \intervals\trials. There is one row for every trial. The ‘id’ column references each trial.

The number of pictures for encoding in each trial is referred to as “load” and is specified in the column “loads” (1–3). The columns loadsEnc1_PicIDs, loadsEnc2_PicIDs, and loadsEnc3_PicIDs specify the identity of the images shown in the encoding stage of each trial. This value can range from 1–5 and is an index into the set of images shown to the subject (see **NWB File Content: Stimulus Group**). If an encoding period did not occur during the trial, the value for that load was set to 0 (i.e., for a trial of load 2, the value in loadsEnc3_PicIDs for that row is = 0). The column ‘loadsProbe_PicIDs’ indicates the identity of the image shown during the probe stage of a trial. The column ‘probe_in_out’ provides the ground truth for the correct answer (1 = in, 0 = out). The ‘response_accuracy’ column denotes whether the answer given by the subject was correct or not (1 = correct, 0 = incorrect).

For each trial, timestamps are provided that can be used for later analysis. All timestamps are in seconds relative to an arbitrary starting point. These values are derived from the raw event TTLs (see **NWB File Content: Acquisition**). The ‘timestamps_FixationCross’ column contains the times of the fixation cross (baseline) onsets. The columns’ timestamps_Encoding(1–3)’ and ‘timestamps_Encoding(1–3)_end’ indicate the start and end times of the encoding periods. If an encoding period did not occur during a given trial, the value for that timestamp was set to 0. The ‘timestamps_Maintenance’ column indicates the time of the maintenance period onset. The ‘timestamps_Probe’ column contains the times of the probe image onset. The ‘timestamps_Response’ column contains the times at which the subject pressed a button on the response pad following probe onset. To conform to NWB: N’s TimeIntervals neurodata type, the required columns ‘start_time’ and ‘stop_time’ have been added and contain each trial’s start/stop times. These values are identical to those specified in ‘timestamps_FixationCross’ and ‘timestamps_Response,’ respectively.

### NWB File content: stimulus group

The \stimulus group contains the images presented to the subjects and the order and time of image presentation. All images shown in the session are saved as a collection of images in the stimulus\templates\StimulusTemplates subgroup. The subgroup stimulus\presentation\StimulusPresentation contains a series of indexes to the StimulusTemplates subgroup in the order in which the images were presented in the session. Each index has an accompanying timestamp that indicates the onset time when this image was presented. In the Sternberg task, to maintain a number of 4 images referenced per trial, a null image (ID = 5, indexed from 0–5) is referenced when an encoding stage did not occur in a given trial due to the load being 1 or 2. The timestamps associated with each null image are saved as an offset of 10 ms from the previous timestamp to maintain a strictly increasing set of timestamps. In the screening task, a null image was not included in ‘StimulusTemplates’ or referenced in ‘StimulusPresentation.’

### NWB File content: units group

The \units group contains all the neuronal data (sorted single neurons). The data in \units is stored in a column-based Dynamic table, with a row for each neuron. The ‘id’ column denotes the cell number. The ‘electrodes’ column indicates the recording electrode to which the cell belongs and contains indexes for the electrode ID, contained in general\extracellular_ephys\electrodes (see **NWB File Content: General Group**). The ‘spike_times_index’ column contains indexes to ‘spike_times,’ a region-based jagged array much larger than the number of rows in the units table, as it is a concatenation of all spike times across all cells. Spike times are specified in units of seconds relative to an arbitrary starting value that is the same across all timestamps in each file. Each entry of ‘spike_times_index’ indexes the last spike of each neuron. Using this referencing scheme, the range of spikes for each neuron can be determined by using the value in ‘spike_times_index’ as the ending value and + 1 the value in ‘spike_times_index’ as the starting index to the subsequent range of spikes.

Additionally, raw waveforms for each spike are provided to facilitate spike waveform feature analysis^48,49^. They are stored in the region-based jagged array ‘waveforms,’ like ‘spike_times’ and ‘spike_times_index,’ but are doubly indexed per NWB standards. For example, the ‘waveforms_index_index’ variable denotes the number of SU waveform indices and waveforms that were detected across a singular electrode. In this case, SUs were solely recorded from one electrode each, so the ‘waveforms_index_index’ vector equals the range from 1 to the number of SUs in the session.

Furthermore, metrics related to the quality of these clusters’ waveforms are provided, such as the waveform mean, waveform standard deviation, isolation distance, the mean projection distance, the mean signal-to-noise ratio, and the signal-to-noise ratio at the mean waveform’s peak. These have been stored as ‘waveform_mean,’ ‘waveform_sd’ ‘waveforms_isolation_distance’, ‘waveforms_mean_proj_dist’, ‘waveforms_mean_snr’, and ‘waveforms_peak_snr’ respectively.

## Technical Validation

Each subject performed two different tasks: the screening task, followed by the working memory task. The data for these two tasks is processed separately and is provided as separate files (see Table 1). One patient (ID 19) did not perform the screening task and only the Sternberg task is provided for this subject.

### Behavior

During the working memory task (Fig. 1b), subjects answered on average 88.73% of trials correctly (Fig. 1d). Accuracy and reaction time varied as a function of load as expected (Fig. 1c), with higher loads resulting in slower and less accurate answers (F_2,40_ = 7.92, P = 0.0013, repeated-measures ANOVA of median reaction times). We note that there was no order recall component at the end of the trial, which is unlike the original Sternberg task^26^. Therefore, in load 3 trials, correctly performing the task required only remembering the identity but not the order of the stimuli shown during encoding. It remains an open question whether the behaviour and/or neural results shown here would differ if the order were required to be remembered^27,50^.

### Data recorded and spike sorting quality metrics

**Fig. 3:**
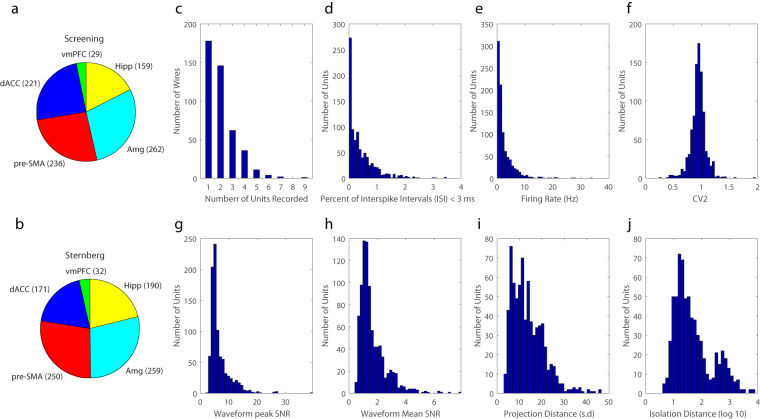
Recording yield and spike sorting quality metrics. (**a**) Number of units isolated in each brain area during the screening task. See Fig. 1e legend for definition of abbreviatons of brain areas used. (**b**) Number of units isolated in each area during the Sternberg task. For panels **a** and **b**, the colors assigned to each region are identical to the assignments in Fig. 1a. (**c**–**j**) Spike sorting metrics for single units recorded in the Sternberg task. (**c**) Number of units recorded on each electrode. (**d**) Percentage of inter-spike intervals (ISIs) that were less than 3 ms. (**e**) Mean firing rates for all units. (**f**) Coefficient of variation (CV2^52^) values for all units. (**g**) Signal-to-noise ratio (SNR) of the peak of the mean waveform (**h**) Mean SNR for all values in a unit’s mean waveform. (**i**) Pairwise projection distance between each unit in which multiple units were found on the same electrode. (**j**) The isolation distance (scaled to log_10_ for ease of viewing) across all units for which this metric was defined.

In the screening task, we recorded 907 neurons (Fig. 3a). Of these, 449 were recorded in the MTL. In the Sternberg task, we recorded a total of 902 neurons (190 hippocampus, 259 Amygdala, 171 dorsal anterior cingulate cortex, 250 pre-supplementary motor area, 32 ventromedial prefrontal cortex) (Fig. 3b). We note that in our prior work, we have not analysed vmPFC neurons but these are provided here for completeness. We note that the two tasks were sorted separately, which is why the numbers of isolated neurons vary slightly. However, based on similarity of selectivity, we typically assume that many of these cells are identical across the two sessions (screening was typically recorded in the morning, followed by Sternberg in the afternoon a few hours later).

We evaluated the quality of the recording and spike sorting using a series of spike sorting quality metrics (see **Methods**). On average across all neurons in the Sternberg task, (*i*) refractory period violations were 0.41% ± 0.50% of interspike intervals (ISIs) (Fig. 3d), (ii) average firing rate was 2.55 Hz, with a range of 0.11–33.82 Hz (Fig. 3e), (*iii*) the pairwise projection distance in clustering space between neurons isolated on the same wire was 13.77 ± 7.32 (projection test; in units of s.d. of the signal)^51^ (Fig. 3g) (*iv*) the ratio between the s.d. of noise and the peak mean waveform amplitude was 6.62 ± 3.63 (Fig. 3g,h), (v) the modified coefficient of variation of the ISIs (CV2^52^) was 0.94 ± 0.14 (Fig. 3f), and (vi) median isolation distance^53^ was 31.14 (Fig. 3j). The isolation distance was calculated in a ten-dimensional feature space (energy, peak amplitude, total area under the waveform, and the first five principal components of the energy normalized waveforms^53^).

### Proportion of selective cells

In this section, we report the proportion of selected concept, maintenance, and probe cells to validate that the exported data reproduces earlier published results^15,20^. All results reported here are directly computed based on the data contained in the provided NWB files. We note that more data is included here, making the results different from those previously published.

**Fig. 4:**
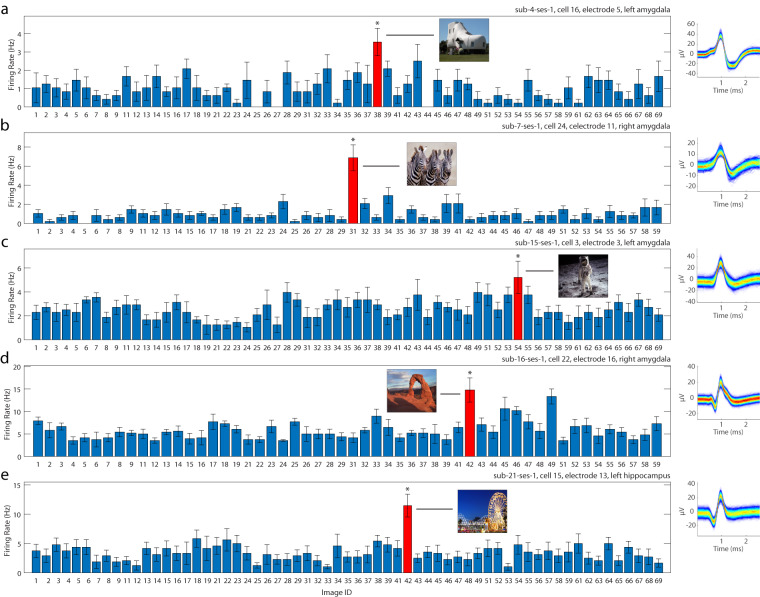
Example neurons identified in screening task. Shown are five (**a**–**e**) neurons classified as concept cells. The image ID specified in the x axis corresponds to the image name stored in the NWB file (see **NWB File Content: Stimulus Group**). The error bars denote the s.e.m. of the firing rate for the 6 instances for which each image was shown. The image to which the cell responded the most strongly is shown in red together with the image shown. The inset on the right shows the spike probability density function across all waveforms associated with the cell. The text label identifies the session and electrode the cell was recorded in.

Screening task: Of the MTL cells, 138 (32.78%) qualified as concept cells (Fig. 4 shows examples). In contrast, only 26 (5.35%) of cells in the MFC were concept cells, reproducing our earlier finding that concept cells are largely only found in the MTL.

**Fig. 5:**
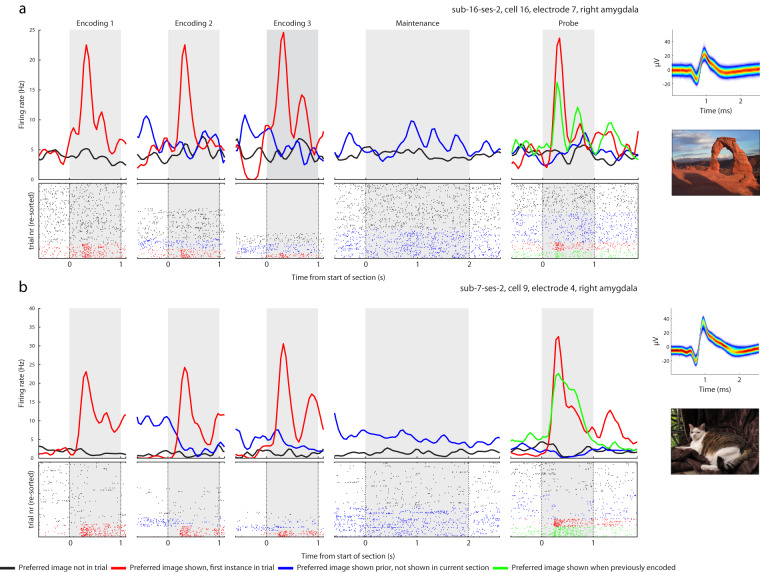
Example neurons identified in the Sternberg task. (**a,b**) Example responses from 2 neurons classified as concept cells in the Sternberg task that also exhibited persistent activity during the maintenance period. Both cells were recorded in the amygdala. Top: peristimulus time histograms of all trial stages (bin size, 50 ms; gaussian smooth width, 75 ms). Note that any applied smoothing is for illustrative purposes only. Insets: spike probability density function across all waveforms associated with the cell and the image to which that cell was selective. Bottom: raster plot across all trials, reordered according to trial condition for visualization purposes. Colors indicates the trial conditions: red for periods of time during which the preferred image was shown the first time in a trial, blue for periods of time during which the preferred image was shown previously in the same trial but not the current time period, green when the preferred image is being repeated after being shown in prior encoding stages, and grey for trials where the preferred image was not displayed. The text label identifies the session and electrode the cell was recorded in.

Working memory task: In the MTL, 94 (20.94%) neurons qualified as concept cells during the encoding period (Fig. 5 shows examples). As a group, concept cells continued to fire during the maintenance period if their preferred stimulus was held in WM (4.76 ± 4.71 Hz vs. 2.67 ± 3.66 Hz; p = 1.38 × 10^−21^ for pref. vs. non-pref. trials, paired one-sided t-test). In the MFC, 144 (31.79%) cells qualified as maintenance cells and 122 (26.93%) as probe cells. These numbers are comparable to those reported in our earlier studies^15,20^.

## Usage Notes

### Timestamps

All timestamps provided in this dataset are in units of seconds relative to the start of the experiment.

### Patient health information

Some data has been omitted or modified to protect patient health information (PHI) as required. For example, ‘session_start_time’ is set to the year the session occurred, with the month/day set to January 1^st^ for all subjects.

### Description of code provided

We provide example code in Matlab to illustrate how to use the data. This code reproduces the figures in this paper and replicates prior results regarding the proportion of selective neurons and spike sorting quality metrics.

### Compatibility of data & code provided

We used MatNWB version 2.6.0.2 for the export and sample import. File validation was performed through Python using the following packages: dandi version 0.55.1, nwbinspector version 0.4.28, & PyNWB version 2.3.1. We tested our analysis code with MATLAB releases 2019, 2022 & 2023 on windows.

## Data Availability

All code associated with this project is available as open source. The code is available on GitHub (https://github.com/rutishauserlab/workingmem-release-NWB). MATLAB scripts are included in this repository to reproduce all figures shown and to illustrate how to use the data.
